# Synthesis and Characterization of Polyaniline Emeraldine Salt (PANI-ES) Colloids Using Potato Starch as a Stabilizer to Enhance the Physicochemical Properties and Processability

**DOI:** 10.3390/ma17122941

**Published:** 2024-06-15

**Authors:** Soufiane Boudjelida, Xue Li, Souad Djellali, Giampiero Chiappetta, Francesca Russo, Alberto Figoli, Mauro Carraro

**Affiliations:** 1 Department of Chemical Science, University of Padova, Via F. Marzolo 1, 35131 Padova, PD, Italy; xue.li.1@studenti.unipd.it; 2 Institute on Membrane Technology, CNR-ITM, UoS of Padova, Via F. Marzolo 1, 35131 Padova, PD, Italy; 3 Institute on Membrane Technology, CNR-ITM, Via P. Bucci 17/C, 87036 Arcavacata di Rende, CS, Italy; g.chiappetta@itm.cnr.it (G.C.); f.russo@itm.cnr.it (F.R.); a.figoli@itm.cnr.it (A.F.); 4 Laboratory of Physical Chemistry of High Polymers, University Ferhat Abbas Setif 1, Setif 19000, Algeria; souad.djellali@univ-setif.dz; 5 Department of Chemistry, Faculty of Sciences, University Ferhat Abbas Setif 1, Setif 19000, Algeria

**Keywords:** starch, polyaniline, conductive, colloids, membranes

## Abstract

Conductive polymers, such as polyaniline (PANI), have interesting applications, ranging from flexible electronics, energy storage devices, sensors, antistatic or anticorrosion coatings, etc. However, the full exploitation of conductive polymers still poses a challenge due to their low processability. The use of compatible stabilizers to obtain dispersible and stable colloids is among the possible solutions to overcome such drawbacks. In this work, potato starch was used as a steric stabilizer for the preparation of colloidal polyaniline (emeraldine salt, ES)/starch composites by exploiting the oxidative polymerization of aniline in aqueous solutions with various starch-to-aniline ratios. The polyaniline/starch bio-composites were subjected to structural, spectroscopic, thermal, morphological, and electrochemical analyses. The samples were then tested for their dispersibility/solubility in a range of organic solvents. The results demonstrated the formation of PANI/starch biocomposites with a smaller average size than starch particles, showing improved aqueous dispersion and enhanced solubility in organic solvents. With respect to previously reported PANI-EB (emeraldine base)/starch composites, the novel colloids displayed a lower overall crystallinity, but the conductive nature of PANI-ES enhanced its electrochemical properties, resulting in richer redox chemistry, particularly evident in its oxidation behavior, as observed through cyclic voltammetry. Finally, as proof of the improved processability, the colloids were successfully integrated into a thin polyether sulfone (PES) membrane.

## 1. Introduction

Conducting polymers (CPs) are difficult to process due to their insolubility in most common solvents [1], which, together with infusibility, limits their potential applications [2,3]. Since colloidal dispersions are frequently employed in place of solutions, developing CP dispersions is a typical method of overcoming this issue [1,4]. To prepare suitable dispersions of CP composites, a steric stabilizer can be added during the chemical oxidative polymerization of the corresponding monomer in an aqueous medium [5,6,7].

As a matter of fact, dispersion polymerization has been used to produce particles with sub-micrometer size [8], whereby the stabilizer in the reaction medium prevents the CP from precipitating and promotes a steady dispersion of particles [1,9]. In this way, colloidal forms of CP, with improved processability, can be prepared [10].

Water-soluble polymers, including cellulose derivatives [6,8], poly(vinyl alcohol) [11], poly(ethylene oxide) [2], and poly(methyl vinyl ether) have been employed as steric stabilizers for CPs [3]. Among CPs, polyaniline (PANI) is one of the most studied, due to its low production cost, mechanical and chemical stability, and adjustable conductivity. Indeed, PANI is the most adaptive conductive polymer, as it occurs in diverse structural forms, and its large range of applications is subsidized by its good redox exchange properties [12,13]. However, PANI is not soluble in most common solvents and displays infusibility, like most CPs [2,3]. The synthesis of new colloidal composites is thus appealing to improve its processability and expand the range of applications [14,15,16].

Previous studies on the preparation of PANI colloids included the use of poly(methyl ethacrylate) [17], poly(vinyl alcohol) [18], and poly(N-vinylpyrrolidone) [8,19]. Many studies also exploited the use of biopolymers, due to their desirable biocompatibility [20,21,22]. In particular, polysaccharide derivatives [6,23,24] were applied to the synthesis of colloidal PANI. Among the others, chitosan [25,26], cellulose [27], nanocellulose [28], and pectin [29,30] were used as steric stabilizers, as they are biodegradable and may impart biological activity.

Increasing attention is now placed on the addition of bio-based compounds derived from agricultural wastes, such as rice husk and walnut shell particles, to polymeric composites [31,32,33]. The convenience is found in the fact that these biodegradable components may improve the thermal, tribological, and physico-mechanical characteristics of the composites and can be easily and inexpensively acquired from agricultural wastes [34]. Starch is a polysaccharide featuring branched linear structures (amylopectin) and linear structures (amylose) produced by higher plants with the role of storing energy in the form of chemical bonds. It demonstrates some suitable physical attributes, being colorless, non-toxic, and biodegradable, making it an environmentally friendly choice compared to synthetic and isotropic stabilizers [35,36,37]. Although it can be isolated from roots and tuber crops (tapioca and potatoes) and cereals (rice, corn, rye, and wheat), it can also be obtained as a byproduct of potato processing industries, making it a sustainable and economical choice for composite preparation. Moreover, its abundance ensures consistent supply, which is crucial for large-scale applications [38]. The molecular structure and functional properties of potato starch, including its film-forming ability, viscosity, and moisture absorption capacity, make it conducive to effective interaction and compatibility with many polymers. This lead to enhanced performance of polymer matrices in applications such as coatings, adhesives, and composites. Previous reports described the use of PANI/starch composites to prepare functional materials. The ability of starch to dissolve and disperse PANI is due to its hydrophilic nature, which can enhance the compatibility between PANI and water-based systems, expanding the possibilities to design novel materials for diverse applications (electrochemical, water treatment, biomedical, etc.) [39,40,41,42,43].

As far as the optimization of the methodologies to obtain polymeric colloids is concerned, this is affected by the reaction medium and the nature of the stabilizing agent used during their synthesis [44,45]. Understanding how these variables influence properties like conductivity, morphology, and stability provides clarity and insight into the strategic choices to drive material design and synthesis methods for the desired specific applications. In this study, polymerization of PANI/starch was performed at 20 °C in an acidic medium, starting with aniline as a monomer, ammonium persulfate (APS) as an oxidative agent, and starch as a stabilizer. The average diameter of the particles was controlled by varying the amount of aniline and starch. Moreover, the conditions were adjusted to promote the formation of conductive emeraldine salt (PANI-ES) [46]. Indeed, the latter displayed intrinsically higher processability, improved stability and biocompatibility, and better control over the electrical conductivity of the polymer [47]. The PANI-ES/starch composites were characterized to establish composition, size, and stability, and one of them was successfully used as a dopant for polyether sulfone (PES) membranes. Characterizations of membrane thickness, pore size, porosity, water contact angle (WCA), and FTIR were conducted on PES membranes with and without PANI-ES/starch composites to assess the effects of the additive on membrane morphology and physico-chemical properties.

## 2. Experimental

### 2.1. Materials

Polyether sulfone (PES) was obtained from Solvay Specialty Polymers (Bollate, Italy). Polyethylene glycol (PEG), polyvinylpyrrolidone (PVP), N-methyl-2-pyrrolidone (NMP), dimethyl sulfoxide (DMSO), N,N-dimethylformamide (DMF), hydrochloric acid, ethanol, methanol, acetone, glycerol, ethyl acetate, hexane, chloroform, cyclohexane, toluene, and ammonium persulfate (APS) were obtained from Sigma Aldrich (Schnelldorf, Germany).

### 2.2. Synthesis of Polyaniline-Starch Particles (PANI-ES/Starch)

Aniline was polymerized in aqueous starch dispersions to produce three PANI/starch materials (Table 1). The procedure [48] involved the addition of aniline at room temperature (20 °C) to an aqueous mixture comprising different wt. ratios of dispersed starch in 1 M HCl, followed by stirring for 4 h. An APS solution was then used to promote the chemical polymerization of aniline, controlling the temperature of the mixture upon immersion in an ice bath (T ~ 0–4 °C). To allow most of the aniline monomers to react, the mixture was kept under magnetic stirring for 3 h. After that, it was left undisturbed for 48 h. The resulting black–greenish powder was then filtered and washed with acetone and water to remove residual reactants and byproducts with low molecular weight, like aniline dimers and oligomers, and finally dried for 2 days under air at 40 °C.

### 2.3. Characterizations of the Materials

A series of spectroscopic characterizations were carried out to assess the successful use of starch as a steric stabilizer: (i) a Nicolet 5700 spectrometer Thermo Fisher equipped (Madison, WI, USA) with sampling accessory for the powdered material (Diamond ATR) was used for FTIR-ATR spectroscopy, (ii) solutions prepared by dissolving 2 mg in 10 mL of DMF were monitored by UV-visible spectroscopy with a Cary 5000 UV-vis-NIR spectrometer (Varian, Palo Alto, CA, USA), and (iii) to finalize the spectral characterization, Raman spectroscopy measurements, using a excitation laser with λ= 532 nm, were carried out with a DXR2 Raman spectrometer (Thermo Scientific, Madison, WI, USA). Thermal analyses were performed with a TGA Q5000 instrument (TA Instruments, Hüllhorst, Germany) with the following setup: heating ramp of 10 °C/min until 700 °C, under nitrogen atmosphere, while DSC analyses were performed with a TA instrument Q20 under a nitrogen atmosphere from room temperature to 300 °C, using a heating ramp of 10 °C/min; data processing and calculations of thermodynamic parameters were carried out using TA Universal Analysis software, version 4.5A. Solubility tests were performed in different solvents and at different temperatures, preparing dilute mixtures (0.2% *w*/*v*) in 10 mL of each solvent. The solvents used were water, ethanol, methanol, acetone, glycerol, ethyl acetate, hexane, chloroform, cyclohexane, toluene, DMF, DMSO, and N-methyl pyrrolidone (NMP) at T = 20, 40, 50, 60, and 80 °C. For insoluble combinations, colorless supernatants were observed; for partly soluble mixtures, a lens was used to detect residual particles. The average particle size was obtained using a laser granulometry analyzer, CILAS 1190 (CPS Us, Inc., Madison, WI, USA). CuKα radiation (λ = 1.54 Å) (X’Pert3 Powder equipment) 2θ range (10° to 60°), at a scan rate of 0.02 s^−1^, was utilized for X-ray diffraction (XRD) examinations. The data were processed utilizing X’pert HighScore Plus Version 3. Using Quanta 200 equipment (FEI, Hillsboro, OR, USA), SEM images of the composites were collected on metallized materials with sputter quorum Q 150R E settings. Three electrodes were used in cyclic voltammetry (CV) testing (using a PGZ 301 potentiometer from Radiometer Analytical SAS, Lyon, France): a reference electrode (Ag/AgCl), an auxiliary platinum electrode, and carbon as the working electrode. A 1 M H_2_SO_4_ solution served as the electrolyte, and voltammograms were collected within a voltage range between −0.8 and +0.8 mV at a scan rate of 100 mV s^−1^.

### 2.4. Membrane Preparation

The phase inverse methodology was implemented to prepare the membranes, which consisted of the immersion of the casting solution in a non-solvent coagulation bath (non-solvent induced phase separation, NIPS). The casting solution was prepared by adding 1 wt.% of Z_11_ dopant to NMP solution containing polyethylene glycol (PEG) and polyvinylpyrrolidone (PVP) as pore-formers [48,49]. PES was dissolved in NMP (Table 2), and the suspension was immersed in an ultrasonic bath (for 90 min at room temperature). Then, it was kept under stirring until a homogeneous casting suspension was observed overnight. The dope solution was cast on a glass plate using a manual casting knife (gap set at 250 µm) at a temperature of around 25 °C. The prepared membranes were washed consecutively with hot water (T=60 °C) to remove traces of pore-formers (PEG and PVP) and dried at room temperature for 4 h. Then, the membranes containing PANI-ES/starch colloids were placed in 0.01 M HCl for 2 h to fix the oxidation state of polyaniline (emeraldine salt form). The membranes were washed with water till neutrality and then dried in an oven at 40 °C for 24 h.

### 2.5. Characterization of the Membranes

The membranes were characterized in terms of thickness, pore size, porosity, contact angle, FTIR, and SEM to evaluate the influence of dopants on the PES membranes. The thickness was measured by a digital micrometer (Mitutoyo Corp. Japan) and registered in 10 regions of each membrane. Then, the average thickness was calculated. The porosity was measured using the gravimetric method, considering the weight of membrane samples before and after the immersion in kerosene (with density ~0.82 g/cm^3^) for around 24 h. The porosity was calculated based on the following equation (1) [49]:

(1) $$Porosity\;\%=\frac{\left(W_{w}-W_{d}\right)/0.82}{\left(W_{w}-W_{d}\right)/0.82+W_{d}/1.37}\times 100$$

where $W_{w}$ is the weight of the wetted membrane, $W_{d}$ is the weight of the dry membrane, 0.82 g/cm^3^ is the density of kerosene, and 1.37 g/cm^3^ is the density of membrane material, which is a calculated statistic number. The pore size of the membranes (mean flow pore size diameter, MFD) was evaluated using the wet–dry curve method by Porometer Porolux^TM^ 1000, Berlin, Germany. Before each test, the membrane was immersed in fluorinated liquid (Porefil^TM^) for ten minutes. The maximum pressure applied to each measurement was 5 bar. The water contact angle (WCA) was measured by the optical contact angle (Meter 200, KSV instruments LTD from Helsinki, Finland) using ultrapure water droplets (5 µL). For each membrane, five measurements were carried out, and the average and standard deviations were calculated. The morphology was determined by SEM (Zeiss EVO Kloten, Swiss). The cross-section samples were fractured in liquid nitrogen and fitted vertically on a sample holder. All samples (top, bottom, and cross sections) were coated with gold by a sputtering machine (Quorum Q 150R S, San Jose, CA, USA). The FTIR was measured using the Spectrum One System by Perkin Elmer Instruments (Waltham, MA, USA). 

## 3. Results and Discussion

### 3.1. Particle Morphology and Dimensions

SEM images of polyaniline/starch composites with increasing aniline-to-starch ratios, shown in Figure S1, show that, when aniline is polymerized in the presence of starch, acting as a template, the polyaniline chains are grafted or adsorbed on the surface [50]. With respect to PANI alone, the size of the PANI/starch granules is smaller, being lower than 50 μm. The rough surface of the composites demonstrates that aniline polymerization occurs on the surface of the starch granules, generating an uneven coating [39,48]. Once the aniline content increases, the starch surface becomes more coated, and polyaniline build-up is observed over the starch particles [42].

**Figure 1:**
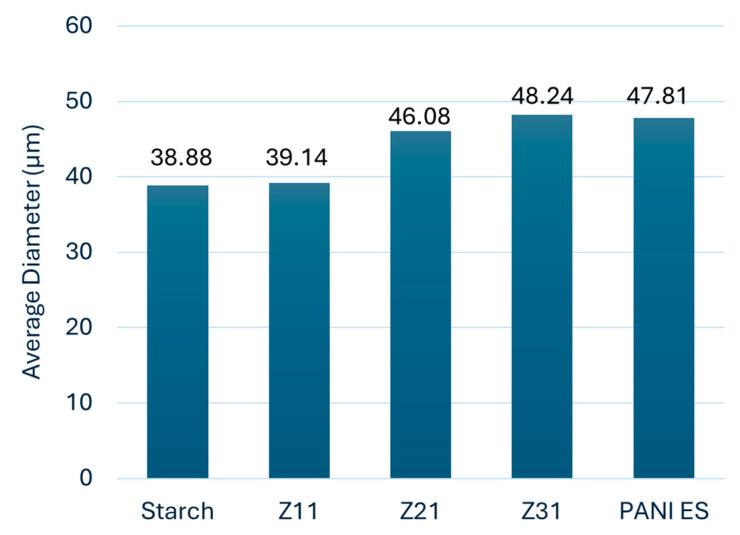
Histogram of the average diameter of the particles (average values of 3 measurements).

Laser granulometry analysis of PANI/starch materials reveals a wide range of particle sizes, indicating high polydispersity and potential particle aggregation [50,51,52,53]. As depicted in Figure 1, colloidal particle diameters range from 39.14 μm to 48.24 μm. These sizes exceed those of starch alone (38.88 μm), suggesting that a higher concentration of aniline in the reaction medium promotes the growth of longer polyaniline chains on the starch particle surface, resulting in larger particles, as also observed in SEM images (Figure S1).

### 3.2. Solubility

The solubility of the materials was tested at various temperatures. Table S1 lists the solvents’ (water, methanol, ethanol, acetone glycerol, ethyl acetate, chloroform, hexane, cyclohexane, toluene, DMF, DMSO, and NMP) ability to dissolve PANI-ES, starch, and PANI-ES/starch composites. PANI-ES is soluble in DMF but insoluble in a wide range of common solvents, while starch is soluble in DMSO but only slightly soluble in water and DMF [20]. We discovered that PANI-ES/starch biocomposites had enhanced dispersion in water at 20–40 °C as well as favorable solubility in DMF, NMP, and DMSO, which deviated from the solubility behavior of homo-polymers, making it possible to obtain clearer mixtures at higher temperatures (60–80 °C). Solubility in glycerol and chloroform was also considerably improved, primarily at higher temperatures (>40 °C), with good dispersions at lower temperatures. The PANI-ES/starch biocomposites could not be dissolved or even dispersed by the remaining solvents. These findings show how the surfaces of the products have distinct affinities for each solvent at different temperatures.

### 3.3. FTIR-ATR

**Figure 2:**
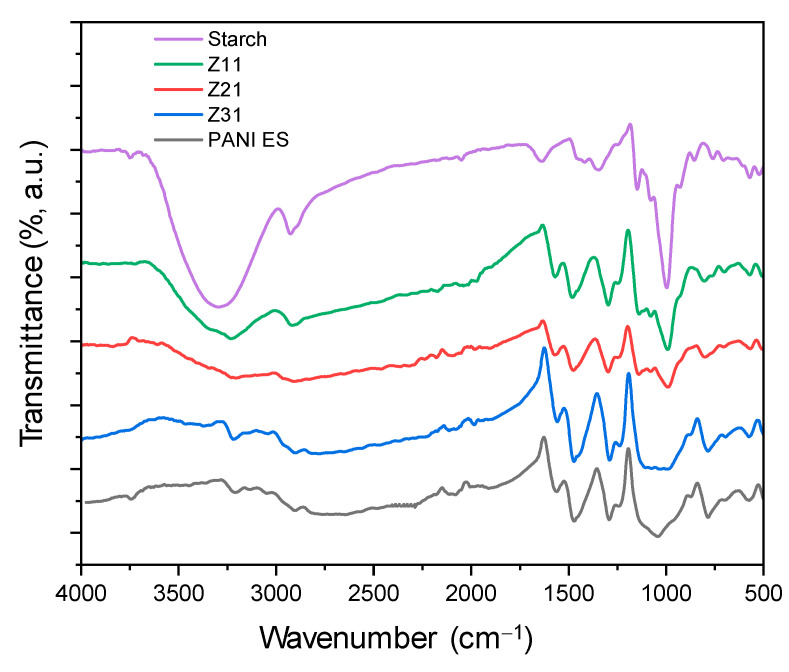
FTIR-ATR spectroscopy spectra of PANI-ES, starch, and PANI-ES/starch biocomposites.

Figure 2 shows the FTIR-ATR spectra of starch, polyaniline, and their biocomposites. The faint peak characteristic of the asymmetric stretching of the carbon–hydrogen (C–H) bond in pyranoid rings is detected at 2924 cm^−1^, whereas the broadband characteristic of starch O–H groups is observed at 3252 cm^−1^. Adsorption bands between 854 cm^−1^ and 1415 cm^−1^ are ascribed to C–H bending and C–O(H) and C–O–C stretching [48,54]. The bands at 1539 cm^−1^ and 1456 cm^−1^ correspond to the vibration of quinoid rings (N=Q=N) and benzenoid rings (N–B–N), while the bands at 1282 cm^−1^ and 1230 cm^−1^ correspond to aromatic C–H bending vibrations. For the composites, PANI-ES signals [55] dominate the spectra, in agreement with efficient surface coverage [56]. The increasing ratio of PANI, from Z_11_ to Z_31_, is clearly highlighted by the decreasing intensity of the starch’s signal at 993 cm^−1^ (C–O stretching) with respect to PANI’s at 1456 cm^−1^ (C–N stretching).

### 3.4. UV-Vis

**Figure 3:**
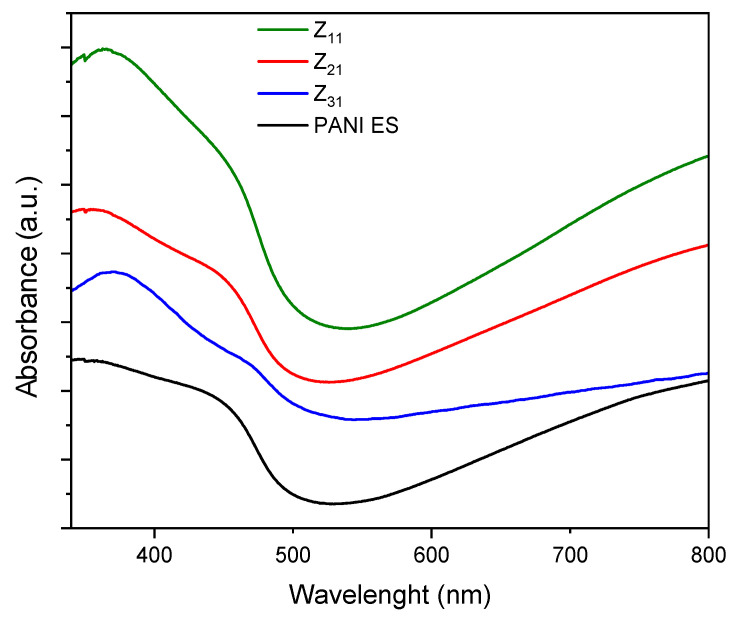
UV-vis spectra of PANI and PANI/starch biocomposites in DMF. Spectra are stacked on an arbitrary scale for clarity reasons.

Figure 3 illustrates the UV-vis spectra of PANI-ES/starch biocomposites [55].

Polyaniline absorption bands match those reported in the literature, where PANI species exhibit two significant absorption bands, one at 250–450 and a broader one between 500 and 900 nm. The first band corresponds to the π–π* transition, whereas the second is related to the transition of benzenoid rings into quinoid rings [57,58]. Other studies correlate these bands to the π–π* transition of benzenoid rings, localized polarons, and delocalized polarons [50,59,60]. These peaks demonstrate that PANI is in an oxidized state, i.e., the emeraldine salt form. In addition, for the composites, the band around 370 nm may be attributed to bond conjugation. Additional changes, with respect to the PANI spectrum, are likely related to the interaction between PANI and starch, together with a variation in the quantity of quinoid and benzenoid units present in the materials.

### 3.5. RAMAN

**Figure 4:**
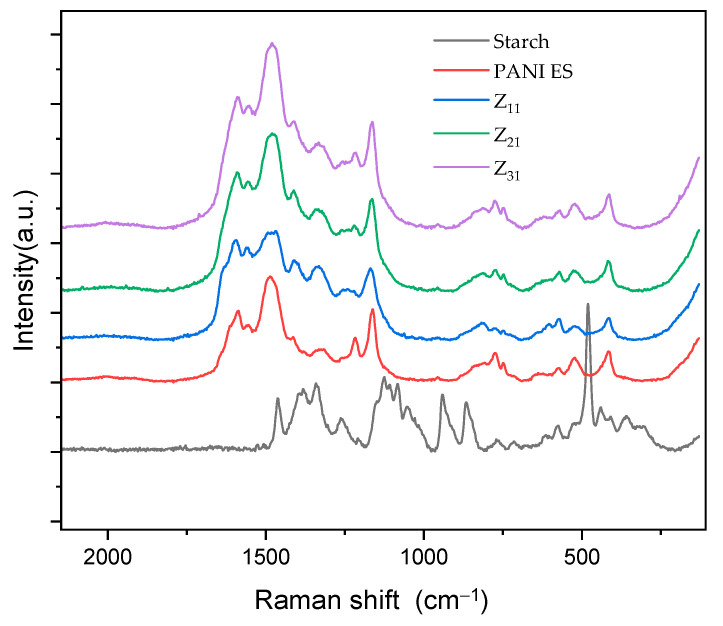
RAMAN spectra of PANI, starch, and PANI/starch biocomposites.

Figure 4 shows the Raman spectra of PANI (ES), starch, and their biocomposites, which were acquired using an excitation wavelength of 532 nm.

The Raman spectra of the composites illustrate the typical signals of PANI [50,59,60] whose principal bands, at 1590 and 1558 cm^−1^, correspond to quinine-type rings and carbon–carbon (ring’s single and double band stretching vibrations), respectively [61,62]. Additionally, distinct bands caused by imine’s C=N stretching modes at 1482 cm^−1^ and the amine group’s C-N stretching at 1221 cm^−1^ can be detected. Raman spectra show the stretching vibrations of an intermediate C~N^+^ bond at a characteristic frequency of approximately 1330 cm^−1^, for which there is no agreement on the exact value of this frequency in the literature [63,64]. N–H bending vibrations are represented by the band at 1562 cm^−1^, while the in-plain deformation mode of quinonoid C–H bonds is observed at 1166 cm^−1^. For PANI chains, other small vibration modes have been observed, such as the band at 777 cm^−1^, which most likely corresponds to quinine ring vibrations. In addition, there are bands occurring at approximately 745 and 809 cm^−1^, which probably correspond to amine and imine deformations [64,65]. Certain vibration modes exhibit variation in intensity and frequency between samples, which can be attributed to factors such as synthetic and doping methodologies, as well as to structural imperfections in the polymer [66].

The way PANI/starch biocomposites respond to laser stimulation is further proof that starch grains are coated by a layer of PANI chain [50], which prevents the laser beam from reaching the inner starch macromolecules.

### 3.6. X-ray Diffraction Pattern (XRD)

**Figure 5:**
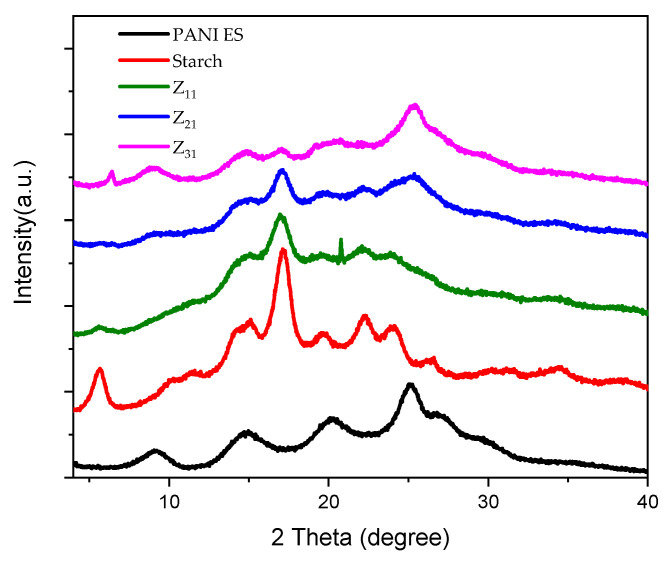
XRD diffractograms of starch, PANI-ES, and PANI-ES/starch biocomposites.

Figure 5 displays the X-ray diffraction patterns of polyaniline, starch, and PANI/starch biocomposites. The semi-crystalline structure of emeraldine salt (ES) is reflected by several wide reflection peaks in the XRD profile of PANI-ES, which represents the crystalline sections and is found at 2θ = 14.9°, 20.21°, and 25.2° [67]. In addition, a semi-crystalline pattern for potato starch can be seen, with weak reflections at 2θ = 15.2° and 19.6°, moderate reflections at 2θ = 5.7°, 15.0°, and 22.2°, and 24.1°, and strong reflections at 2θ = 17.2°. B-type crystalline starch structures, commonly referred to as tuber starches, typically exhibit this pattern [50,68].

Bragg’s equation is used for each peak to calculate the interplanar distance “d”:

nλ = 2d sinθ, (2)

where θ is the Bragg angle for the matching peak, λ = 1.542 Å, and n = 1 (first-order reflection). Table 3 shows our materials’ diffraction spacing and Miller indices (hkl).

The ratio of the percentage of the peak’s crystalline area to the overall area of the XRD pattern beneath the curve yielded the degree of crystallinity (X_c_%) values. The following Formula (3) is used [69]:

X_c_ (%) = H_c_/(H_c_ + H_a_) *×* 100, (3)

where Hc is the intensity of crystalline profiles, and Ha is the intensity of amorphous profiles.

Table 4 summarizes the crystallinity degree values of starch, polyaniline, and biocomposites. Compared to polyaniline (19%), native starch exhibits a higher degree of crystallinity (37%). For PANI–starch colloids, a contribution due to different starch content can be assessed. A shift in the crystallinity may cause changes in XRD patterns, which might indicate modifications in the ultrastructure of the PANI and starch microdomains. The shift of PANI-ES peaks and the disappearance of starch peaks at 2θ = 22.2° and 26.6° complement this observation. It appears that the original crystalline structure of starch is partially destroyed by the presence of aniline in the reaction media and PANI chains during propagation. This is especially true for Z_21_ and Z_31_.

### 3.7. Thermal Characterization

**Figure 6:**
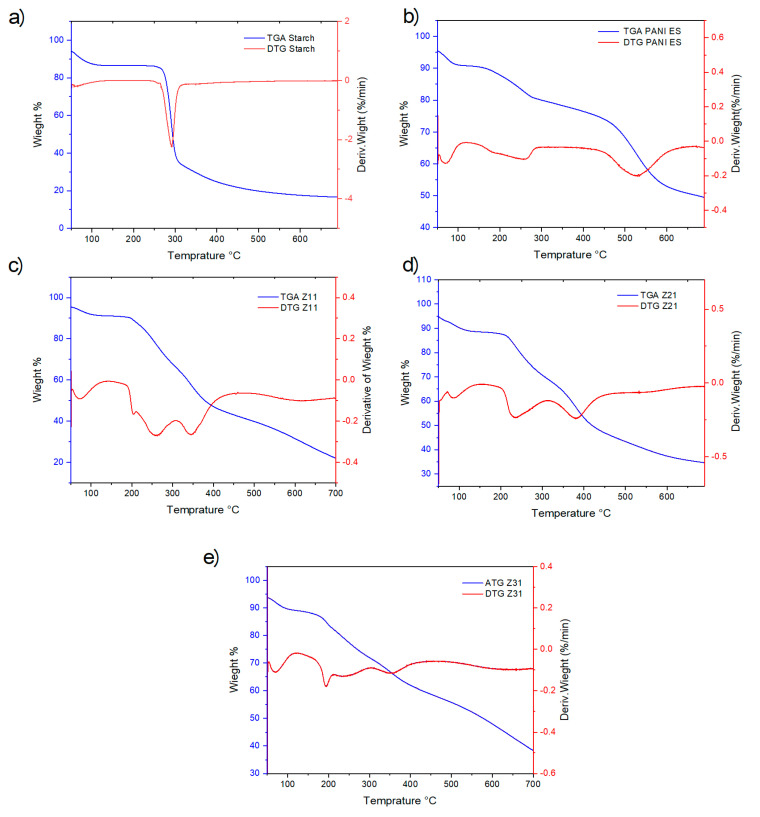
TGA and first derivative DTG curves for starch (**a**), PANI-ES (**b**), and their blends: Z_11_ (**c**), Z_21_ (**d**), and Z_31_ (**e**).

The TG/DTG curves resulting from the thermal gravimetric analysis of PANI, starch, and their biocomposites are depicted in Figure 6, which illustrates a sharp step indicative of starch degradation and a broad temperature range for weight loss of PANI. At 290 °C, native starch does, in fact, lose 50% of its weight (Figure 6a and Table 5), while PANI exhibits a corresponding decrease in weight at 673 °C (Figure 6b and Table 5). In detail, PANI-ES displays an initial weight loss occurring between 50 °C and 100 °C, most likely due to the removal of volatile molecules and evaporation of water. Additionally, a small endothermic peak is observed between 210 °C and 250 °C. Subsequently, significant weight loss occurs from 400 °C onwards due to the degradation of PANI chains [54].

The DTG curves show that the biocomposites’ overall degradation rate decreases as their aniline content increases, underscoring the significant influence of polyaniline on their thermal stability (Figure 6c,d). It is also noteworthy that the distinctive decomposition pattern of PANI, highlighted by a prominent DTG peak at 500 °C, is no longer clearly evident for the composites.

TGA thermograms, which are associated with thermal stability, also provide the values for T5, T50, and T95, which are the temperatures at which 5, 50, and 95% of the mass is volatilized, respectively, as illustrated in Table 5. Biocomposites and PANI-ES are noticeably more stable than starch.

**Figure 7:**
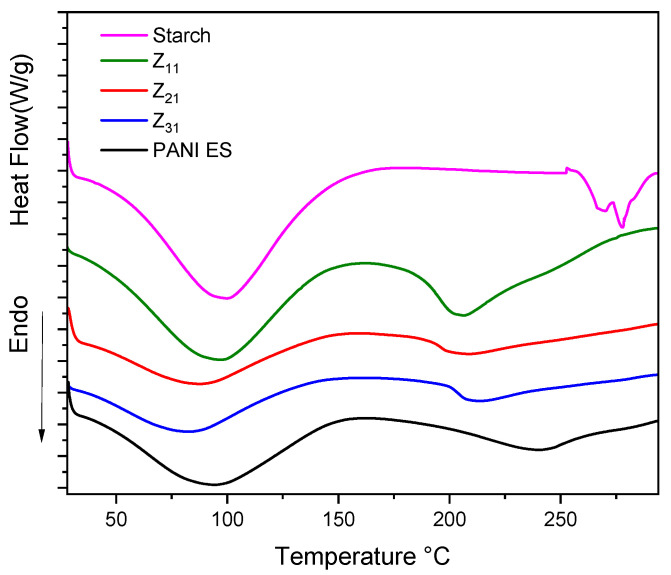
DSC thermograms curves of starch, PANI-ES, and Z_11_, Z_21_, and Z_31_ biocomposites.

Figure 7 presents the DSC profiles of starch, polyaniline, and their biocomposites. Broad endothermic peaks between 60–140 °C are attributed to water evaporation, consistent with TGA and DTG data. PANI thermograms display a second endothermic peak at 240 °C, potentially indicating chain segment mobility and glass transition temperature (Tg) [70,71]. Starch thermograms exhibit two narrow endothermic peaks at 266 °C and 278 °C, reflecting its thermal breakdown [72], absent in the composites, in line with TGA analysis. In PANI/starch materials, the water evaporation peak is evident, and the second endothermic peak shifts to a lower temperature, especially for Z_11_, possibly due to component interactions, confirming structural changes in the biocomposites (for detailed DSC data, refer to Table 5).

### 3.8. Cyclic Voltammetry

**Figure 8:**
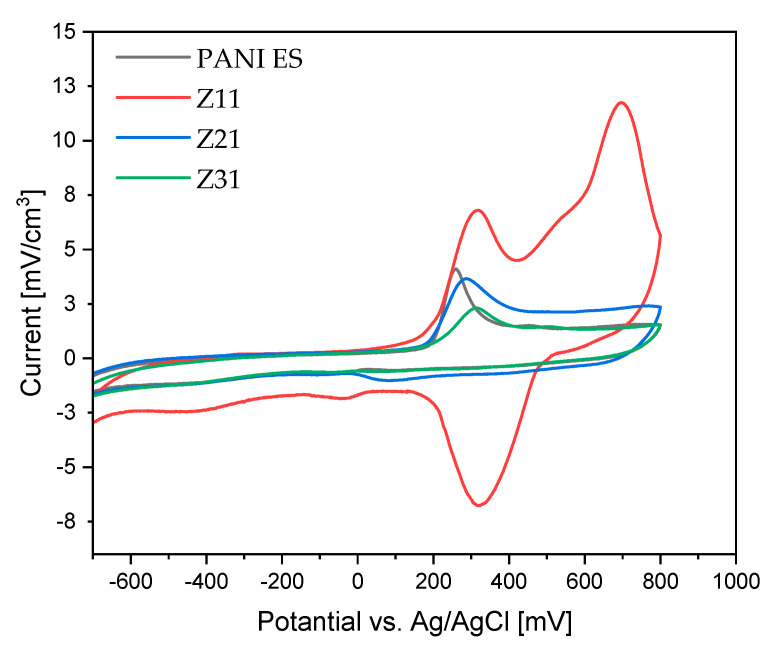
Cyclic voltammograms of PANI and PANI/starch biocomposites, measured at 100 mV s^−1^ using an Ag/AgCl reference electrode in H_2_SO_4_ solution (1 M).

On the CV curve (Figure 8), two pairs of anodic and cathodic current peaks indicate two distinct sets of redox activity (Table 6). The first pair of redox couple peaks appears between 0 and 0.25 V vs. Ag/AgCl reference electrode, and represents the conversion of the completely reduced leucoemeraldine base to partly oxidized emeraldine. The redox current peaks in the second set, which occur between 0.6 and 0.8 V vs. Ag/AgCl, are related to the conversion of emeraldine to the fully oxidized pernigraniline form. While the second redox couple (peaks Ox2 and Red1) potential is highly reliant on pH, the first redox couple (peaks Ox1 and Red2) potential is mostly independent of pH [73]. Since no proton is involved, this latter reduction reaction (Equation (4)) may be defined as follows: [74].



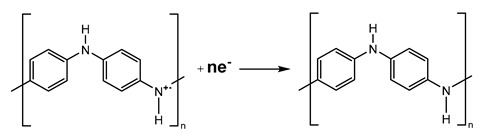

(4)


However, the pH of the solution has a significant impact on the peak position of the second redox process, so it is reasonable to presume that protons are involved in the reaction illustrated below (Equation (5)).



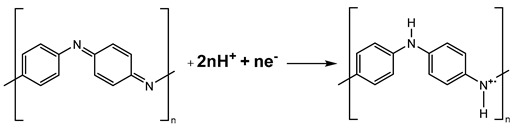

(5)


Notably, when the scan rate value increases (see Figure S2 and Table S3 in the Supporting Information), the current density increases as well, demonstrating the materials’ strong electro-activity and electrochemical capacitive behavior [72].

All PANI/starch synthesized by chemical oxidation polymerization are conducting materials, thus representing an advancement with respect to previously reported PANI-emeraldine base/starch composites [50]. Among the colloids, the Z_11_ sample is the most electroactive material, showing the highest current for both oxidation and reduction processes in the explored range.

### 3.9. Embedding of PANI-ES/Starch into a Polymeric Membrane

To demonstrate the easy processability of the biocomposites, the Z_11_ sample was used to prepare a thin membrane based on polyether sulfone (PES). In order to make the membrane porous, a mixture of PEG (21 wt.%) and PVP (5 wt.%) was added to the blend as a pore former. The membranes were prepared from NMP solutions using the phase inversion technique upon immersion in a water coagulation bath after casting (thickness set at 250 μm) on a glass surface [75]. The membranes were characterized in terms of FTIR, WCA, porosity, pores size surface, and cross-section SEM images.

**Figure 9:**
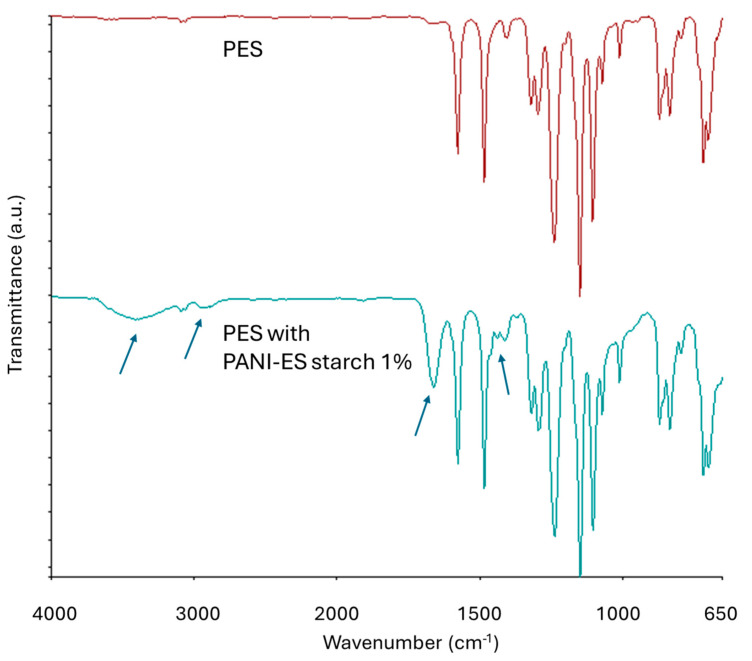
ATR-FTIR of the PES membranes with and without 1% Z_11_ dopants.

The membranes’ ATR-FTIR spectra are presented in Figure 9.

The investigation indicates that 1% dopants have little effect on the surface composition of membranes, which displays the signal pattern of the PES matrix (stretching vibrations can be observed at 1578 cm^−1^ for the aromatic rings, 1240 cm^−1^ for the ether linkages, C–O–C bonds, 1150 and 1105 cm^−1^ for the sulfone groups). There are additional peaks, indicated by arrows in Figure 9, for the PES membrane with Z_11_, which are likely ascribed to O–H (3420 cm^−1^), C–H (2917 cm^−1^), and C–O (1665 cm^−1^) bonds of starch (see also Figure S3 in the Supporting Information) [59].

WCAs were measured on the top side of the PES membrane in order to investigate their surface properties, and the results are shown in Table 7. WCAs range from 72° to 75°, showing the hydrophilic properties of both membranes, with negligible effect of the dopant.

**Figure 10:**
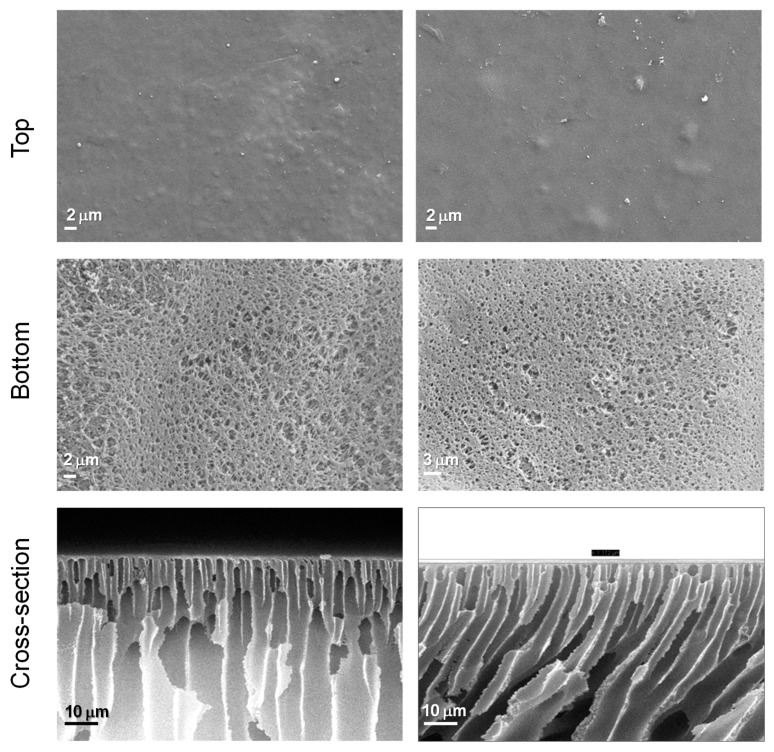
SEM images (top, bottom, and cross-section) of the PES membranes with and without 1% Z_11_ dopants.

SEM was used to obtain further insights into membrane features (see images in Figure 10, which collects images of the cross-section, top, and bottom surface of the prepared membranes).

From the SEM images, we observe that there is no agglomeration of Z_11_ or phase separation (with the exception of some spots on the membrane surface), which is evidence of the miscibility of the PANI starch colloid blended in the PES matrix. Both membranes (with and without dopant) exhibit macro-porous asymmetric structures. The asymmetry of the membranes consist of a dense top (or up) layer and a porous sub-layer (bottom) [76]. For PES, the dense layer is supported by a region with finger-like type channels of ca. 0.9 μm diameter, with an overall thickness of approximately 153 μm and overall porosity of roughly 90%. For the composite membranes, the denser layer is also connected to regular finger-like macro-channels, with diameters of ca. 0.13 μm. The opposite side is characterized by the presence of even larger voids of a sponge-like region, confined by a bottom surface with narrower openings (diameter < 3 μm). The impact of the dopants on the membranes’ thickness is more evident, being increased from 153 μm to 228 μm. This is probably due to the addition of dopants, that enhance the demixing of polymers and non-solvents, leading to faster coagulation of the membrane. This behavior also favors the formation of smaller macro voids and has an impact on the overall porosity decrease [77], being appealing to increase selectivity and rejection during separation processes. All in all, these results are interesting for the development of conductive membranes for water filtration, which will be the object of future investigation. Indeed, conductive membranes are promising for the possibility of tuning selectivity, imparting antifouling properties, and promoting electrochemical processes such as electrocoagulation or electrooxidation. For example, conductive polymers can enhance the membrane’s filtration efficiency by attracting and repelling charged particles in water. This can lead to better removal of contaminants such as heavy metals, bacteria, and organic compounds such as ionic dyes [78].

## 4. Conclusions

The outcomes of this work show how to easily synthesize PANI-based colloids. Specifically, potato starch was utilized as a steric stabilizer to produce stable dispersions of polyaniline (emeraldine salt). Thus, polyaniline/starch biocomposites with varying ratios of aniline to starch were produced via oxidative polymerization.

Starch acts as a template, leading to improved solubility of polyaniline in DMSO, NMP, and DMF while improving its higher-temperature dispersion in glycerol, water, and chloroform. This, in turn, is expected to enhance polyaniline’s processability. In order to assess the impact of variations in the aniline/starch ratios, several tests, including cyclic voltammetry and FTIR-ATR, Raman, UV-Vis, XRD, TGA, and DSC, were carried out.

Surface analyses show that polyaniline chains grow on the surface of starch granules. The degree of crystallinity and particle size exhibit significant changes in the composite material, as evidenced by the growth of smaller particles and the loss of the native starch crystalline lattice. Despite being less thermally stable, Z_11_ appears to be the one with a better morphology and degree of crystallinity. In terms of electron exchange and capacitive behavior, cyclic voltammetry tests demonstrate improved electro-activity in the composite materials, confirming Z_11_ as the best formulation.

As far as processability is concerned, a hydrophilic PES membrane was prepared with the highly dispersed Z_11_. The latter had an effect on membrane morphology (thickness, pore size, and shape). In particular, the addition of a dopant decreased the overall pore size and increased the membrane thickness while maintaining the same hydrophilicity, making itpromising for applications in the field of water filtration.

The obtained composites were demonstrated to be polyaniline colloids with appealing features, namely conductivity, biocompatibility, and processability. The polyaniline/starch composites are expected to be adaptable materials that will increase the range of uses for colloidal PANI. These may include the following: (i) use as conductive coatings on various substrates such as plastics, glass, and textiles, with applications in antistatic coatings, electromagnetic shielding, and corrosion protection [79]; (ii) incorporation into chemical sensors and smart devices for environmental monitoring and healthcare diagnostics [80]; (iii) preparation of electrode materials, where they can enhance the energy storage capacity and cycling stability of supercapacitors and batteries, due to their high capacitance and electrochemical stability [81].

## Figures and Tables

**Table 1:** Names of the prepared samples and their aniline:starch wt ratio.

Aniline: Starch wt ratio	1:1	2:1	3:1
Codes	Z_11_	Z_21_	Z_31_

**Table 2:** Components of the casting solutions.

	PES (wt.%)	Z_11_ (wt.%)	PVP (wt.%)	PEG (wt.%)	NMP (wt.%)
PES-0	12	0	5	35	48
PES-1	12	1	5	35	47

**Table 3:** Calculated diffraction spacing “d” and Miller indices of starch and PANI-ES.

Product	2θ (°)	hkl	d (Å)
PANI-ES	14.9	(011) 010	5.95
	20.2	(020) 100	4.40
	25.2	(200) 111	3.53
Starch	5.7	(001)	15.51
	11.4	(111)	7.76
	15.0	(140)	5.89
	17.3	(131)	5.14
	19.6	(103)	4.53
	22.2	(113)	4.00
	24.1	(132)	3.69
	26.6	(142)	3.35

**Table 4:** Degree of crystallinity of starch, PANI-ES, and their biocomposites.

Material	Degree of Crystallinity (%)
Starch	37.0
Z_11_	35.4
Z_21_	33.6
Z_31_	22.8
PANI-ES	19.6

**Table 5:** DSC thermogram curves, elaborated by TA Universal Analysis software of starch, PANI-ES, and the biocomposites Z_11_, Z_21_, and Z_31_.

Material	1st Peak			2nd Peak		
	Peak (°C)	Onset (°C)	∆H (j/g)	Peak (°C)	Onset (°C)	∆H (j/g)
Z_11_	95.9	42.7	234.5	207.8	186.7	103.6
Z_21_	84.4	37.8	287.3	217.6	199.9	95.2
Z_31_	98.9	45.8	247.6	205.4	168.4	212.9
PANI-ES	98.1	35.3	275.1	218.4	199.6	23.3
				293.5	281.2	1.1
Starch	100.0	50.5	439.7	266.4	261.7	9.1
				278.1	273.8	15.8

**Table 6:** Current density measured at 100 mV s^−1^ against an Ag/AgCl reference electrode in 1 M H_2_SO_4_ solution from the cyclic voltammogram peaks of PANI-ES and the PANI-ES/starch biocomposites.

Products	Ox 1	Ox 2	Red 1	Red 2
Z_11_	6.80	11.75	−6.77	−1.80
Z_21_	3.63	2.41	−0.69	−0.77
Z_31_	2.30	1.47	−0.47	−0.64
PANI-ES	4.11	1.50	−0.37	−0.69

**Table 7:** Characteristics of the prepared PES membranes with and without 1% Z_11_ dopants.

Z_11_ Content in Membranes	WCA (Top Surface) (°)	Thickness (µm)	Porosity (%)	Mean Flow Pore Size Diameter (MFD) (µm)
0%	72 ± 3	153 ± 6	91 ± 0	0.9
1%	75 ± 2	228 ± 7	90 ± 0	0.13

## Data Availability

The raw data supporting the conclusions of this article will be made available by the authors on request.

## Supplementary Materials

The following supporting information can be downloaded at: https://www.mdpi.com/article/10.3390/ma17122941/s1, Table S1: Solubility of PANI-ES/Starch composites; Table S2: Elaborated thermal characteristics (% weight loss at specific temperatures) from TGA curves; Table S3. Redox parameters from cyclic voltammogram peaks of PANI and PANI/starch bio-composites; Figure S1. SEM images of (a): Starch, (b) PANI-ES and PANI-ES/starch biocomposites: (c): (1:1), (d): (2:1) and (e): (3:1). Figure S2. Cyclic voltammograms of PANI and PANI/starch biocomposites; Figure S3. FTIR-ATR of PES and PES with PANI-ES/starch 1%wt.

## References

[1] Marcilla R., Ochoteco E., Pozo-Gonzalo C., Grande H., Pomposo J.A., Mecerreyes D. (2005). New Organic Dispersions of Conducting Polymers Using Polymeric Ionic Liquids as Stabilizers. Macromol. Rapid Commun..

[2] Cawdery N., Obey T.M., Vincent B. (1988). Colloidal Dispersions of Electrically Conducting Polypyrrole Particles in Various Media. J. Chem. Soc. Chem. Commun..

[3] Digar M.L., Bhattacharyya S.N., Mandal B.M. (1992). Conducting Polypyrrole Particles Dispersible in Both Aqueous and Non-Aqueous Media. J. Chem. Soc. Chem. Commun..

[4] Stejskal J., Sapurina I. (2005). Polyaniline: Thin Films and Colloidal Dispersions (IUPAC Technical Report). Pure Appl. Chem..

[5] Lu Y., Pich A., Adler H.P. (2004). Synthesis and Characterization of Polypyrrole Dispersions Prepared with Different Dopants. Macromol. Symp..

[6] Mandal T.K., Mandal B.M. (1995). Ethylhydroxyethylcellulose Stabilized Polypyrrole Dispersions. Polymer.

[7] Kwon J.-Y., Kim E.-Y., Kim H.-D. (2004). Preparation and Properties of Waterborne-Polyurethane Coating Materials Containing Conductive Polyaniline. Macromol. Res..

[8] Bjorklund R.B., Liedberg B. (1986). Electrically Conducting Composites of Colloidal Polypyrrole and Methylcellulose. J. Chem. Soc. Chem. Commun..

[9] Stejskal J., Kratochvíl P., Helmstedt M. (1996). Polyaniline Dispersions. 5. Poly(Vinyl Alcohol) and Poly(*N*-Vinylpyrrolidone) as Steric Stabilizers. Langmuir.

[10] Stejskal J., Kratochvíl P., Armes S.P., Lascelles S.F., Riede A., Helmstedt M., Prokeš J., Křivka I. (1996). Polyaniline Dispersions. 6. Stabilization by Colloidal Silica Particles. Macromolecules.

[11] Armes S.P., Miller J.F., Vincent B. (1987). Aqueous Dispersions of Electrically Conducting Monodisperse Polypyrrole Particles. J. Colloid Interface Sci..

[12] Ohsawa T., Kabata T., Kimura O., Nakajima S., Nishihara H., Yoshino K. (1993). Non-Linear Electric Properties of Polyaniline Doped with Organic Acceptors. Synth. Met..

[13] Subathira A., Meyyappan R. (2010). Inhibition of Corrosion of Steel Alloy Using Polyaniline Conducting Polymer Coatings. Int. J. Chem. Sci..

[14] Li Y., Ying B., Hong L., Yang M. (2010). Water-Soluble Polyaniline and Its Composite with Poly(Vinyl Alcohol) for Humidity Sensing. Synth. Met..

[15] Shannon K., Fernandez J.E. (1994). Preparation and Properties of Water-Soluble, Poly(Styrenesulfonic Acid)-Doped Polyaniline. J. Chem. Soc. Chem. Commun..

[16] Tallman D.E., Wallace G.G. (1997). Preparation and Preliminary Characterization of a Poly(4-Vinylpyridine) Complex of a Water-Soluble Polyaniline. Synth. Met..

[17] Winnik M., Lukas R., Chen W., Furlong P. (1987). Studies of the Dispersion Polymerisation of Methyl Methacrylate in Nonaqueous Media. Makromol. Chem. Macromol. Symp..

[18] Arenas M., Sánchez G., Martínez-Álvarez O., Castaño V. (2014). Electrical and Morphological Properties of Polyaniline–Polyvinyl Alcohol in Situ Nanocomposites. Compos. Part B Eng..

[19] Riede A., Helmstedt M., Riede V., Stejskal J. (1998). Polyaniline Dispersions. 9. Dynamic Light Scattering Study of Particle Formation Using Different Stabilizers. Langmuir.

[20] Skotheim T.A., Reynolds J. (2006). Conjugated Polymers: Theory, Synthesis, Properties, and Characterization.

[21] Eisazadeh H., Gilmore K., Hodgson A., Spinks G., Wallace G. (1995). Electrochemical Production of Conducting Polymer Colloids. Colloids Surf. A Physicochem. Eng. Asp..

[22] Bober P., Humpolíček P., Syrový T., Capáková Z., Syrová L., Hromádková J., Stejskal J. (2017). Biological Properties of Printable Polyaniline and Polyaniline–Silver Colloidal Dispersions Stabilized by Gelatin. Synth. Met..

[23] Eisazadeh H., Spinks G., Wallace G. (1994). Electrochemical Production of Polypyrrole Colloids. Polymer.

[24] Kašpárková V., Jasenská D., Capáková Z., Maráková N., Stejskal J., Bober P., Lehocký M., Humpolíček P. (2019). Polyaniline Colloids Stabilized with Bioactive Polysaccharides: Non-Cytotoxic Antibacterial Materials. Carbohydr. Polym..

[25] Cruz-Silva R., Arizmendi L., Del-Angel M., Romero-Garcia J. (2007). pH-and Thermosensitive Polyaniline Colloidal Particles Prepared by Enzymatic Polymerization. Langmuir.

[26] Cruz-Silva R., Escamilla A., Nicho M., Padron G., Ledezma-Perez A., Arias-Marin E., Moggio I., Romero-Garcia J. (2007). Enzymatic Synthesis of pH-Responsive Polyaniline Colloids by Using Chitosan as Steric Stabilizer. Eur. Polym. J..

[27] Chattopadhyay D., Mandal B.M. (1996). Methyl Cellulose Stabilized Polyaniline Dispersions. Langmuir.

[28] Luong N.D., Korhonen J.T., Soininen A.J., Ruokolainen J., Johansson L.-S., Seppälä J. (2013). Processable Polyaniline Suspensions through in Situ Polymerization onto Nanocellulose. Eur. Polym. J..

[29] Amarnath C.A., Venkatesan N., Doble M., Sawant S.N. (2014). Water Dispersible Ag@ Polyaniline-Pectin as Supercapacitor Electrode for Physiological Environment. J. Mater. Chem. B.

[30] Djellali S., Touati A., Kebaili M., Sahraoui R. (2021). Synthesis of Polyaniline/Pectin Biocomposite and Its Efficiency as Adsorbent for Methylene Blue Removal. Recent Advances in Environmental Science from the Euro-Mediterranean and Surrounding Regions.

[31] Aridi N., Sapuan S., Zainudin E., AL-Oqla F.M. (2016). Mechanical and Morphin ological Properties of Injection-Molded Rice Husk Polypropylene Composites. Int. J. Polym. Anal. Charact..

[32] Schirp A., Barrio A. (2018). Fire Retardancy of Polypropylene Composites Reinforced with Rice Husks: From Oxygen Index Measurements and Cone Calorimetry to Large-scale Single-burning-item Tests. J. Appl. Polym. Sci..

[33] Salasinska K., Barczewski M., Górny R., Kloziński A. (2018). Evaluation of Highly Filled Epoxy Composites Modified with Walnut Shell Waste Filler. Polym. Bull..

[34] Singh T., Gangil B., Patnaik A., Biswas D., Fekete G. (2019). Agriculture Waste Reinforced Corn Starch-Based Biocomposites: Effect of Rice Husk/Walnut Shell on Physicomechanical, Biodegradable and Thermal Properties. Mater. Res. Express.

[35] Jiménez A., Fabra M.J., Talens P., Chiralt A. (2013). Physical Properties and Antioxidant Capacity of Starch–Sodium Caseinate Films Containing Lipids. J. Food Eng..

[36] Yan Q., Hou H., Guo P., Dong H. (2012). Effects of Extrusion and Glycerol Content on Properties of Oxidized and Acetylated Corn Starch-Based Films. Carbohydr. Polym..

[37] Azmi N.S., Kadir Bahsa R., Othman S.H., Mohammed M.A.P. (2019). Characterization of Antioxidant Tapioca Starch/Polyaniline Composites Film Prepared Using Solution Casting Method. Food Res..

[38] Torres M.D., Fradinho P., Rodríguez P., Falqué E., Santos V., Domínguez H. (2020). Biorefinery Concept for Discarded Potatoes: Recovery of Starch and Bioactive Compounds. J. Food Eng..

[39] Saikia J.P., Banerjee S., Konwar B.K., Kumar A. (2010). Biocompatible Novel Starch/Polyaniline Composites: Characterization, Anti-Cytotoxicity and Antioxidant Activity. Colloids Surf. B Biointerfaces.

[40] Lukasiewicz M., Ptaszek P., Ptaszek A., Bednarz S. (2014). Polyaniline–Starch Blends: Synthesis, Rheological, and Electrical Properties. Starch-Stärke.

[41] Pandi N., Sonawane S.H., Gumfekar S.P., Kola A.K., Borse P.H., Ambade S.B., Guptha S., Ashokkumar M. (2019). Electrochemical Performance of Starch-Polyaniline Nanocomposites Synthesized by Sonochemical Process Intensification. J. Renew. Mater..

[42] Janaki V., Vijayaraghavan K., Oh B.-T., Lee K.-J., Muthuchelian K., Ramasamy A., Kamala-Kannan S. (2012). Starch/Polyaniline Nanocomposite for Enhanced Removal of Reactive Dyes from Synthetic Effluent. Carbohydr. Polym..

[43] Gautam V., Srivastava A., Singh K.P., Yadav V.L. (2017). Preparation and Characterization of Polyaniline, Multiwall Carbon Nanotubes, and Starch Bionanocomposite Material for Potential Bioanalytical Applications. Polym. Compos..

[44] Hosseinzadeh S., Saadat Y., Abdolbaghi S., Afshar-Taromi F., Hosseinzadeh A. (2014). Shape of the Particles Produced by Seeded Dispersion Polymerization of Styrene. Colloid J..

[45] Cho Y.-S., Shin C.H., Han S. (2016). Dispersion Polymerization of Polystyrene Particles Using Alcohol as Reaction Medium. Nanoscale Res. Lett..

[46] Amalina A.N., Suendo V., Reza M., Milana P., Sunarya R.R., Adhika D.R., Tanuwijaya V.V. (2019). Preparation of Polyaniline Emeraldine Salt for Conducting-Polymer-Activated Counter Electrode in Dye Sensitized Solar Cell (DSSC) Using Rapid-Mixing Polymerization at Various Temperature. Bull. Chem. React. Eng. Catal..

[47] Rai R., Roether J.A., Boccaccini A.R. (2022). Polyaniline Based Polymers in Tissue Engineering Applications: A Review. Prog. Biomed. Eng..

[48] Nazarzadeh Z.E., Najafi M.P., Azariyan E., Sharifian I. (2011). Conductive and Biodegradable Polyaniline/Starch Blends and Their Composites with Polystyrene. Iran. Polym. J..

[49] Russo F., Tiecco M., Galiano F., Mancuso R., Gabriele B., Figoli A. (2022). Launching Deep Eutectic Solvents (DESs) and Natural Deep Eutectic Solvents (NADESs), in Combination with Different Harmless Co-Solvents, for the Preparation of More Sustainable Membranes. J. Membr. Sci..

[50] Boudjelida S., Djellali S., Ferkous H., Benguerba Y., Chikouche I., Carraro M. (2022). Physicochemical Properties and Atomic-Scale Interactions in Polyaniline (Emeraldine Base)/Starch Bio-Based Composites: Experimental and Computational Investigations. Polymers.

[51] Sulimenko T., Stejskal J., Křivka I., Prokeš J. (2001). Conductivity of Colloidal Polyaniline Dispersions. Eur. Polym. J..

[52] Do Nascimento G.M., Silva C.H., Temperini M.L. (2008). Spectroscopic Characterization of the Structural Changes of Polyaniline Nanofibers after Heating. Polym. Degrad. Stab..

[53] Furukawa Y., Ueda F., Hyodo Y., Harada I., Nakajima T., Kawagoe T. (1988). Vibrational Spectra and Structure of Polyaniline. Macromolecules.

[54] Lee D., Char K. (2002). Thermal Degradation Behavior of Polyaniline in Polyaniline/Na+-Montmorillonite Nanocomposites. Polym. Degrad. Stab..

[55] Hou X., Zhou Y., Liu Y., Wang L., Wang J. (2020). Coaxial Electrospun Flexible PANI//PU Fibers as Highly Sensitive pH Wearable Sensor. J. Mater. Sci..

[56] Gautam V., Srivastava A., Singh K.P., Yadav V.L. (2016). Vibrational and Gravimetric Analysis of Polyaniline/Polysaccharide Composite Materials. Polym. Sci. Ser. A.

[57] Laska J., Widlarz J. (2005). Spectroscopic and Structural Characterization of Low Molecular Weight Fractions of Polyaniline. Polymer.

[58] Masters J., Sun Y., MacDiarmid A., Epstein A. (1991). Polyaniline: Allowed Oxidation States. Synth. Met..

[59] Šeděnková I., Trchová M., Stejskal J. (2008). Thermal Degradation of Polyaniline Films Prepared in Solutions of Strong and Weak Acids and in Water–FTIR and Raman Spectroscopic Studies. Polym. Degrad. Stab..

[60] Ćirić-Marjanović G., Trchová M., Stejskal J. (2008). The Chemical Oxidative Polymerization of Aniline in Water: Raman Spectroscopy. J. Raman Spectrosc. Int. J. Orig. Work. Asp. Raman Spectrosc. Incl. High. Order Process. Brillouin Rayleigh Scatt..

[61] Izumi C.M., Brito H.F., Ferreira A.M.D., Constantino V.R., Temperini M.L. (2009). Spectroscopic Investigation of the Interactions between Emeraldine Base Polyaniline and Eu (III) Ions. Synth. Met..

[62] do Nascimento G.M., Silva C.H., Izumi C.M., Temperini M.L. (2008). The Role of Cross-Linking Structures to the Formation of One-Dimensional Nano-Organized Polyaniline and Their Raman Fingerprint. Spectrochim. Acta Part A Mol. Biomol. Spectrosc..

[63] Bober P., Trchová M., Prokeš J., Varga M., Stejskal J. (2011). Polyaniline–Silver Composites Prepared by the Oxidation of Aniline with Silver Nitrate in Solutions of Sulfonic Acids. Electrochim. Acta.

[64] Mažeikienė R., Niaura G., Malinauskas A. (2010). A Comparative Raman Spectroelectrochemical Study of Selected Polyaniline Derivatives in a pH-Neutral Solution. Synth. Met..

[65] Hao Q., Lei W., Xia X., Yan Z., Yang X., Lu L., Wang X. (2010). Exchange of Counter Anions in Electropolymerized Polyaniline Films. Electrochim. Acta.

[66] Abdullah H.S. (2012). Electrochemical Polymerization and Raman Study of Polypyrrole and Polyaniline Thin Films. Int. J. Phys. Sci.

[67] Liu R., Qiu H., Zong H., Fang C. (2012). Fabrication and Characterization of Composite Containing HCl-Doped Polyaniline and Fe Nanoparticles. J. Nanomater..

[68] Colonna P., Buleon A., Merciar C., Galliard T. (1987). Starch: Properties and Potential.

[69] Doumeng M., Makhlouf L., Berthet F., Marsan O., Delbé K., Denape J., Chabert F. (2021). A Comparative Study of the Crystallinity of Polyetheretherketone by Using Density, DSC, XRD, and Raman Spectroscopy Techniques. Polym. Test..

[70] Wei Y., Jang G.-W., Hsueh K.F., Scherr E.M., MacDiarmid A.G., Epstein A.J. (1992). Thermal Transitions and Mechanical Properties of Films of Chemically Prepared Polyaniline. Polymer.

[71] Ding L., Wang X., Gregory R. (1999). Thermal Properties of Chemically Synthesized Polyaniline (EB) Powder. Synth. Met..

[72] Hejna A., Lenża J., Formela K., Korol J. (2019). Studies on the Combined Impact of Starch Source and Multiple Processing on Selected Properties of Thermoplastic Starch/Ethylene-Vinyl Acetate Blends. J. Polym. Environ..

[73] Song E., Choi J.-W. (2013). Conducting Polyaniline Nanowire and Its Applications in Chemiresistive Sensing. Nanomaterials.

[74] Huang W.-S., Humphrey B.D., MacDiarmid A.G. (1986). Polyaniline, a Novel Conducting Polymer. Morphology and Chemistry of Its Oxidation and Reduction in Aqueous Electrolytes. J. Chem. Soc. Faraday Trans. Phys. Chem. Condens. Phases.

[75] Yu J., Boudjelida S., Galiano F., Figoli A., Bonchio M., Carraro M. (2022). Porous Polymeric Membranes Doped with Halloysite Nanotubes and Oxygenic Polyoxometalates. Adv. Mater. Interfaces.

[76] Russo F., Galiano F., Pedace F., Aricò F., Figoli A. (2020). Dimethyl Isosorbide As a Green Solvent for Sustainable Ultrafiltration and Microfiltration Membrane Preparation. ACS Sustain. Chem. Eng..

[77] Wang H.H., Jung J.T., Kim J.F., Kim S., Drioli E., Lee Y.M. (2019). A Novel Green Solvent Alternative for Polymeric Membrane Preparation via Nonsolvent-Induced Phase Separation (NIPS). J. Membr. Sci..

[78] Yang L., Wu S., Chen J.P. (2007). Modification of Activated Carbon by Polyaniline for Enhanced Adsorption of Aqueous Arsenate. Ind. Eng. Chem. Res..

[79] Sharma N., Singh A., Kumar N., Tiwari A., Lal M., Arya S. (2024). A review on polyaniline and its composites: From synthesis to properties and progressive applications. J. Mater. Sci..

[80] Gualandi I., Tessarolo M., Mariani F., Possanzini L., Scavetta E., Fraboni B. (2021). Textile Chemical Sensors Based on Conductive Polymers for the Analysis of Sweat. Polymers.

[81] Li Z., Gong L. (2020). Research Progress on Applications of Polyaniline (PANI) for Electrochemical Energy Storage and Conversion. Materials.

