# Supplementary material for: Synthesis and Characterization of Polyaniline Emeraldine Salt (PANI-ES) Colloids Using Potato Starch as a Stabilizer to Enhance the Physicochemical Properties and Processability
Source: Materials (Basel). 2024 Jun 15;17(12):2941. doi: 10.3390/ma17122941 (PMC11205985; doi:10.3390/ma17122941)
Supplement: Supplementary file 1 [file materials-17-02941-s001.zip › materials-2892626-supplementary.pdf]

Supplementary Information for:

Synthesis and Characterization of Polyaniline Emeraldine Salt (PANI-ES) Colloids Using Potato Starch as a Stabilizer to Enhance the Physicochemical Properties and Processability

Soufiane Boudjelida, Xue Li, Souad Djellali, Giampiero Chiappetta, Francesca Russo, Alberto Figoli, Mauro Carraro

SEM

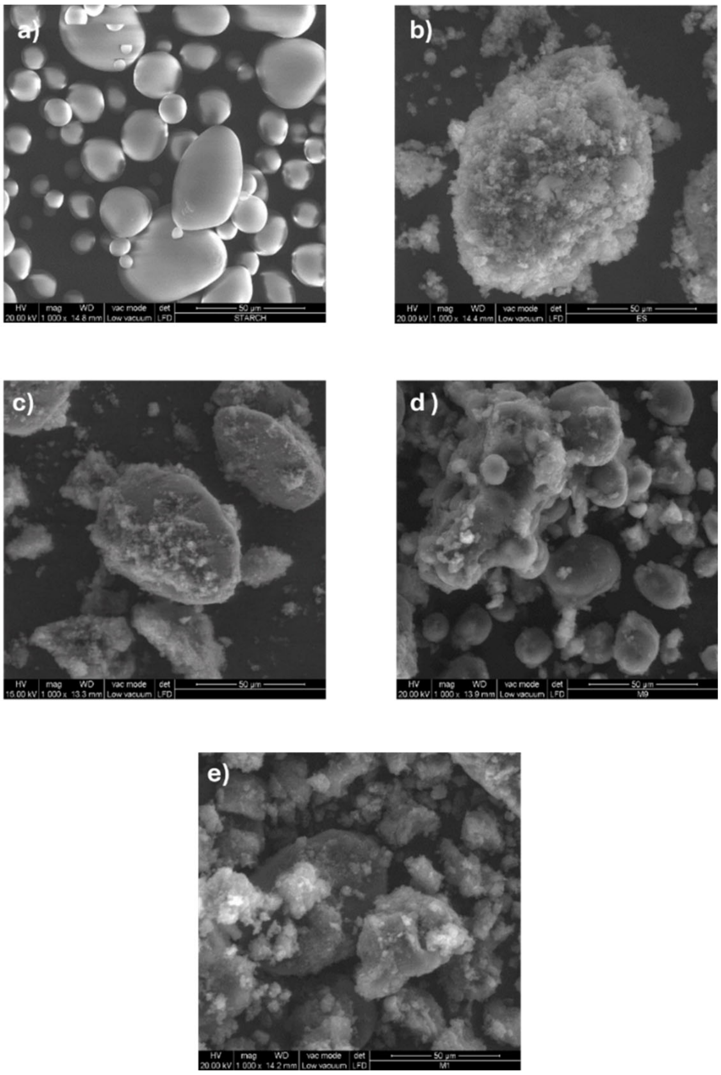

Figure S1. SEM images of (**a**): Starch, (**b**) PANI-ES and PANI-ES/starch biocomposites: (**c**): (1:1), (**d**): (2:1) and (**e**): (3:1).

Solubility data

Table S1: Solubility of PANI-ES/Starch composites.

T°C	ES-Starch (W% w%)	Acetone	Methanol	Ethanol	Glycerol	Ethyle Acetate	Chloroform	Toluene	Hexane	Cyclohexane	DMSO	Water	DMF	NMP
20	Z11	Δ	Δ	Δ	Δ	Δ	Δ	Δ	Δ	Δ	O	Δ	X	O
	Z21	Δ	Δ	Δ	Δ	Δ	Δ	Δ	Δ	Δ	O	Δ	X	O
	Z31	Δ	Δ	Δ	Δ	Δ	Δ	Δ	Δ	Δ	O	Δ	X	O
	PANI ES	Δ	Δ	Δ	Δ	Δ	Δ	Δ	Δ	Δ	O	Δ	X	O
	Starch	Δ	Δ	Δ	Δ	Δ	Δ	Δ	Δ	Δ	X	O	O	X
40	Z11	Δ	Δ	Δ	Δ	Δ	Δ	Δ	Δ	Δ	O	Δ	X	O
	Z21	Δ	Δ	Δ	Δ	Δ	Δ	Δ	Δ	Δ	O	Δ	X	O
	Z31	Δ	Δ	Δ	Δ	Δ	Δ	Δ	Δ	Δ	O	Δ	X	O
	PANI ES	Δ	Δ	Δ	Δ	Δ	Δ	Δ	Δ	Δ	O	Δ	X	O
	Starch	Δ	Δ	Δ	Δ	Δ	Δ	Δ	Δ	Δ	X	O	O	X
60	Z11	-	Δ	Δ	O	Δ	O	Δ	Δ	Δ	X	Δ	X	X
	Z21	-	Δ	Δ	O	Δ	O	Δ	Δ	Δ	X	Δ	X	X
	Z31	-	Δ	Δ	O	Δ	O	Δ	Δ	Δ	X	Δ	X	X
	PANI ES	-	Δ	Δ	Δ	Δ	Δ	Δ	Δ	Δ	X	Δ	X	X
	Starch	-	Δ	Δ	Δ	Δ	Δ	Δ	Δ	Δ	X	O	O	X
80	Z11	-	-	-	O	Δ	-	Δ	Δ	Δ	X	Δ	X	X
	Z21	-	-	-	O	Δ	-	Δ	Δ	Δ	X	Δ	X	X
	Z31	-	-	-	O	Δ	-	Δ	Δ	Δ	X	Δ	X	X
	PANI ES	-	-	-	Δ	Δ	-	Δ	Δ	Δ	X	Δ	X	X
	Starch	-	-	-	Δ	Δ	-	Δ	Δ	Δ	X	O	O	X

Symbols: Δ: not soluble, O: slightly soluble, X: soluble, -: evaporation of the solvents

Table S2: Elaborated thermal characteristics (% weight loss at specific temperatures) from TGA curves. Data were obtained by TA Universal Analysis software of starch, PANI-ES, and the biocomposites, respectively Z_{11}, Z_{21}, and Z_{31}.

Materials	T 5% (°C)	T 10% (°C)	T 50% (°C)	T 95% (°C)	Residual (%) at 700 °C
PANI-ES	56.4	164.2	673.0	--	50
Starch	50.2	70.2	289.6	-	16.5
Z_{11}	59.8	197.1	376.8	--	23.9
Z_{21}	50.3	102.2	416.7	--	35.1
Z_{31}	50.6	91.7	574.3	--	40.7

Cyclic voltammetry

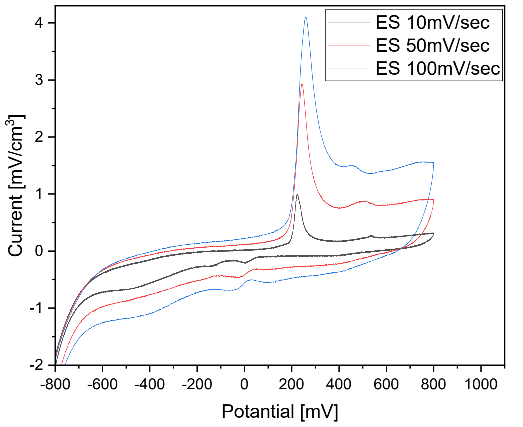

PANI ES

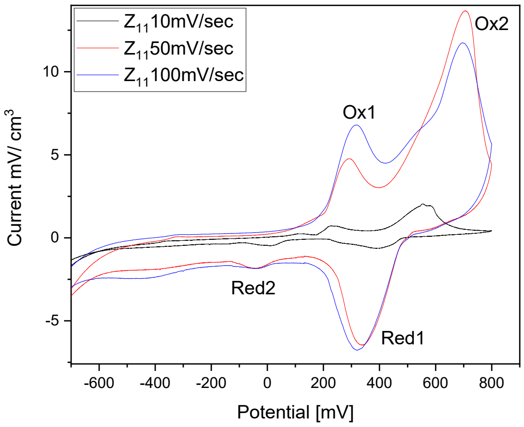

Z_{11}

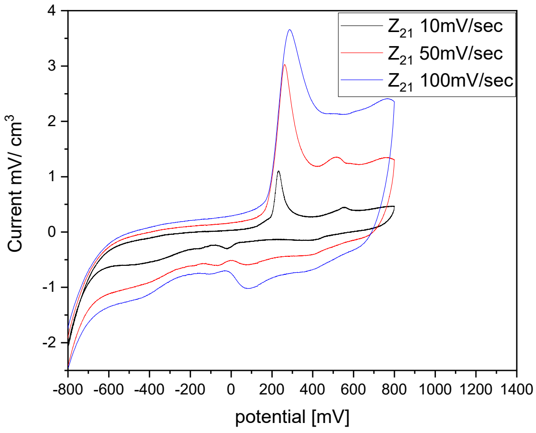

Z_{21}

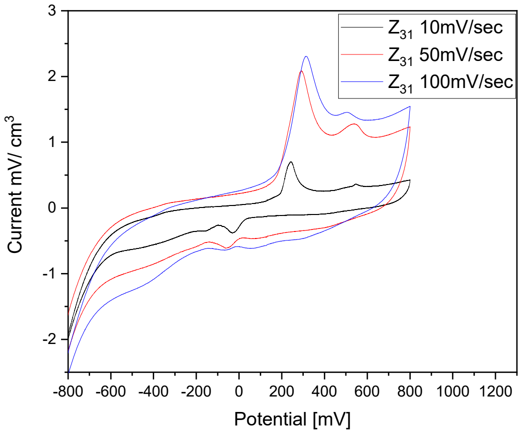

Z_{31}

Figure S2. Cyclic voltammograms of PANI and PANI/starch biocomposites.

Table S3. Redox parameters from cyclic voltammogram peaks of PANI and PANI/starch biocomposites.

Products	Scan rate mV/sec	OX 1	OX 2	Red 1	Red 2
Z_{11}	10	0.71	2.1	-0,71	-0.57
	50	4.7	13.74	-6.43	-1.8
	100	6.8	11.75	-6.77	-1.8
Z_{21}	10	1.1	0.46	-0.17	-0.31
	50	3.03	1.38	-0.41	-0.6
	100	3.63	2.41	-0.69	-0.77
Z_{31}	10	0.71	0.36	-0.09	-0.38
	50	2.08	1.28	-0.31	-0.6
	100	2.30	1.47	-0.47	-0.64
PANI ES	10	0.99	0.26	-0.08	-0.2
	50	2.93	0.88	-0.22	-0.47
	100	4.11	1.5	-0.37	-0.69

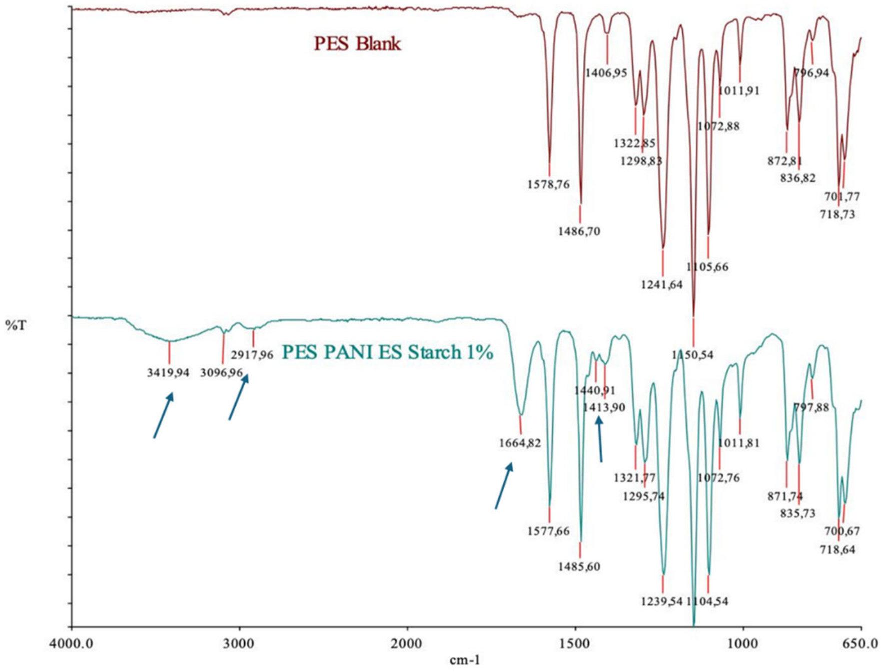

Figure S3. FTIR-ATR of PES and PES with PANI-ES/starch 1%wt. The peaks indicted by arrows are mostly due to starch. Residual PVP can also contribute to the peak at 1665 cm^{-1} (C=O stretching).
